# Applying Differential Wave-Front Sensing and Differential Power Sensing for Simultaneous Precise and Wide-Range Test-Mass Rotation Measurements

**DOI:** 10.3390/s21010164

**Published:** 2020-12-29

**Authors:** Neda Meshksar, Moritz Mehmet, Katharina-Sophie Isleif, Gerhard Heinzel

**Affiliations:** 1 Institut für Geophysik, ETH Zürich, Sonneggstrasse 5, CH-8092 Zürich, Switzerland; 2 Max-Planck-Institut für Gravitationsphysik (Albert-Einstein-Institut) and Leibniz Universität Hannover, Callinstraße 38, 30167 Hannover, Germany; katharina-sophie.isleif@desy.de (K.-S.I.); gerhard.heinzel@aei.mpg.de (G.H.)

**Keywords:** differential wave-front sensing, differential power sensing, deep frequency modulation interferometry, test-mass readout, torsion balance

## Abstract

We propose to combine differential wave-front sensing (DWS) and differential power sensing (DPS) in a Mach-Zehnder type interferometer for measuring the rotational dynamics of a test-mass. Using the DWS method, a high sensitive measurement of 6 nrad Hz^−1/2^ in sub-Hz frequencies can be provided around the test-mass nominal position (±0.11 mrad), whereas the measurement of a wide rotation range (±5 mrad) is realized by the DPS method. The interferometer can be combined with deep frequency modulation (DFM) interferometry for measurement of the test-mass translational dynamics. The setup and the resulting interferometric signals are verified by simulations. An optimization algorithm is applied to find suitable positions of the lenses and the waist size of the input laser in order to determine the best trade of between the slope of DWS, dynamic range of DPS, and the interferometric contrast. Our simulation further allows to investigate the layout for robustness and design tolerances. We compare our device with a recent experimental realization of a DFM interferometer and find that a practical implementation of the interferometer proposed here has the potential to provide translational and rotational test-mass tracking with state-of-the-art sensitivity. The simple and compact design, and especially the capability of sensing the test-mass rotation in a wide range and simultaneously providing a high-precision measurement close to the test-mass nominal position makes the design especially suitable for example for employment in torsion pendulum setups.

## 1. Introduction

High precision displacement measurement of a freely floating test-mass is essential for the space-based gravitational wave detectors, such as the Laser Interferometer Space Antenna (LISA) [1], and also for the accelerometers and the gradiometers applied in the missions like the Gravity Recovery and Climate Experiment (GRACE) [2], its follow on (GRACE-FO) [3], and the Gravity Field and steady-state Ocean Circulation Explorer (GOCE) [4] and their expected successors.

A torsion balance is a powerful tool to develop sensors for precise measurement of the test-mass dynamics. It typically consists of an auto-collimator or capacitive sensors for sensing the rotational and (possibly via a lever arm) the translational test-mass displacement. The torsion pendulum setup at the university of Trento investigated the Gravitational Reference Sensors for the LISA Pathfinder mission [5] by applying an auto-collimator, which has an intrinsic resolution of 20 nrad Hz^−1/2^ above 1 mHz, and the Gravitational Reference Sensors with sensitivity of 2 nm Hz^−1/2^ in translational displacement and 200 nrad Hz^−1/2^ in rotation above 1 mHz. More precise displacement measurement can be provided by applying interferometric readout methods, such as a dual-heterodyne interferometer developed by Hao Yan et al. [6] demonstrating noise levels of approximately 1 pm Hz^−1/2^ and 0.5 nrad Hz^−1/2^ at 1 Hz for linear and angular measurements, respectively. A less sensitive, but compact heterodyne interferometer was developed by Schuldt et al. [7] with noise levels below 10 pm Hz^−1/2^ and 10 nrad Hz^−1/2^ for frequencies $>10^{-2}$ Hz. LISA Pathfinder has demonstrated a sensitivity of 34 fm Hz^−1/2^ above 30 mHz frequencies in orbit using heterodyne laser interferometry [8,9].

Although heterodyne interferometers provide more sensitive measurements compared to other readout methods, their complex design and large scale can be a disadvantage, especially for implementation in space missions. Further investigations tried to design interferometers with a simple design and small scale, such as the one introduced by Isleif et al. [10]. The setup in this proof-of-principle experiment was based on the deep frequency modulation (DFM) interferometry [11] and has achieved a sensitivity of 250 pm Hz^−1/2^ in linear displacement at 1 mHz. Later, they presented a single-component DFM interferometer, which demonstrated tilt and displacement measurements with a precision of less than 20 nrad Hz^−1/2^ and 1 pm Hz^−1/2^ at frequencies below 1 Hz [12]. The DFM signal processing is based on the non-linear fit algorithm originally developed by Heinzel et al. for the deep phase modulation (DPM) interferometry technique [13] with which a sensitivity of 20 pm Hz^−1/2^ in length, and 10 nrad Hz^−1/2^ in angle at millihertz frequencies was demonstrated. The DPM and DFM interferometry methods allow for a simple interferometer design with a highly sensitive translational measurement. The rotation measurements however, can be improved by combining these interferometers with the differential wave-front sensing (DWS) and differential power sensing (DPS) methods, which is the main subject of this paper.

In this paper, we propose a simple and compact implementation of a Mach-Zehnder type interferometer, which measures the test-mass rotation with a potentially achievable sensitivity better than 6 nrad Hz^−1/2^ for a dynamic range of ±0.11 mrad around the test-mass nominal position using the DWS signal. Furthermore, the interferometer measures also a wide-range test-mass rotation of ±5 mrad using the DPS signal. The simple design, the small interferometer scale that requires only a few optical elements, the high sensitivity measurement around the test-mass nominal position, and the wide range measurement using the DPS method make the interferometer design ideally suited for application in torsion pendulum experiments. The proposed interferometer can be combined with the DFM or DPM interferometry for simultaneous sensing of wide range linear and angular displacement. The efficiency of the interferometer is maximized by determining the best waist size of the input laser beam and the position of the applied lenses in the interferometer design. This is achieved by applying an optimization algorithm using our in-house software package for Computer Aided Design of Interferometers, IFOCAD [14]. We then project the measurement results from the IFOCAD simulations onto the measurement results by Isleif et al. [12] to estimate the angular noise for the simulated interferometer.

This paper is structured as follows—Section 2 introduces the interferometer setup and Section 3 elaborates on the applied optimization for obtaining the maximum performance. Furthermore, it presents the optimized setup and the estimated sensitivity. Considerations for a practical implementation are provided in Section 4. Finally, a conclusion is provided in Section 5.

## 2. Interferometer Setup

**Figure 1:**
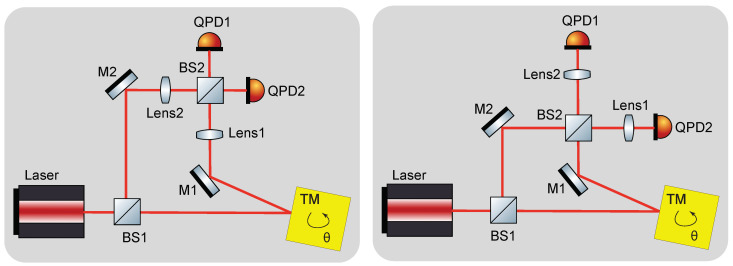
Possible positioning of the lenses in the interferometer setup with the test-mass (TM) in nominal position. (**Left**) lenses implemented before beam-splitter BS2; (**Right**) lenses implemented after BS2. The interferometric signals on the two quadrant photo diodes (QPD) are symmetric, therefore one of them can be considered as a redundant.

Figure 1 illustrates our proposed Mach-Zehnder type interferometer setup. A 50/50 beam-splitter (BS1) splits the laser beams into two sub-beams. One sub-beam serves as a local reference beam while the other one serves as the sensing beam which is sent towards a test-mass (TM). Highly reflective steering mirrors (M1,M2) are placed such that the two sub-beams recombine on a second 50/50 beam-splitter (BS2). To image the sub-beams on the photo diodes, lenses (Lens1, Lens2) are applied. These can be positioned before or after BS2 as shown in the figure. The interferometer arms have unequal length by design. The large distance (a few hundred millimeters) between test-mass and BS1 is especially advantageous for integrating the interferometer into a torsion pendulum setup. Two quadrant photo diodes (QPDs) sense the interferometer output signal. We propose to implement a DFM readout scheme which allows to extract the translation of the test-mass and provides the required signal to sense its rotation via DWS and DPS. For DFM the carrier frequency of the laser source needs to be modulated. The optimal choice for the modulation depth and modulation frequency depend on the arm length mismatch. Typical values are 1–10 GHz for the modulation depth and around 1 kHz for the modulation frequency. An arm length mismatch is essential, since for the DFM interferometry technique the input beam carries the modulation and thus, arms of equal length would lead to a cancellation of the frequency modulation signal. Details on the implementation of a DFM interferometer can be found, for example, in Reference [10,11]. To achieve a simple interferometer setup the number of optical elements is kept minimal, that is, only one lens in each interferometer arm is implemented, instead of using an imaging system that consists of multiple lenses. The interferometric signals on the two photo diodes are symmetric, and thus, one of the photo diodes is redundant and it could be eliminated. Therefore, the setup with lenses after BS2 requires one lens less than the other setup in the minimal configuration. However, for reasons of redundancy and noise reduction it may be advantageous to retain both readout ports. It has been shown that DFM can also be used in a Michelson type interferometer [10]. A Michelson interferometer would also work for rotation measurements. However, in contrast to the Mach-Zehnder topology where the two output signals are readily accessible, retrieving the interferometric signal of the second readout port (the light field reflected back towards the laser source) requires additional polarising optics. Furthermore, applying lenses in a Michelson type interferometer would not be desirable before the beam-splitter, as the double transmission through the lens would result in more stray light.

2 Our setup would also be suited for alternative interferometric techniques such as DPM or heterodyne interferometry which also allow for multi-fringe tracking of the test-mass translation but require different laser preparation. A DPM setup could be realized by using a laser source without frequency modulation and by placing in one arm a piezo-mounted mirror that is driven with a sinusoidal signal to introduce a differential phase modulation. Heterodyne interferometry, which typically employs acousto-optic modulators to frequency-shift the laser beams before interference at the second beam-splitter, could also be used. However, as mentioned above, this typically leads to rather bulky setups which is a disadvantage when aiming for extending the sensing to more degrees of freedom or when the available space is limited.

## 3. Efficiency Optimization and the Interferometer Layout

**Figure 2:**
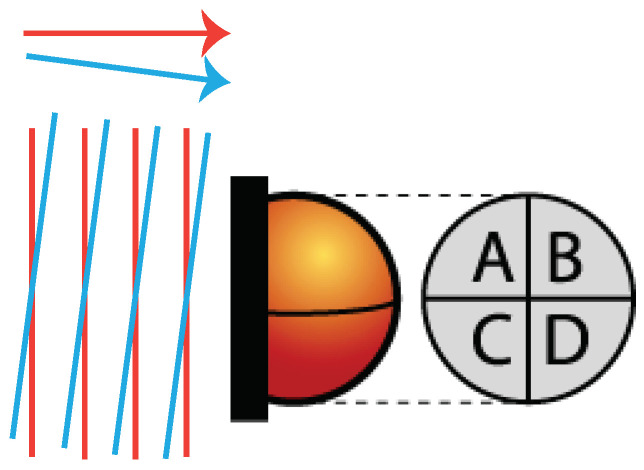
Principle of the differential wave-front sensing. Tilted beams cause phase-shifted beat notes on the different segments of a quadrant photo diode. The tilt angle can be determined by subtracting the phase of beat notes measured on the different photo diode segments.

The highly sensitive rotation measurement is achieved by differential wave-front sensing (DWS) [15,16]. Figure 2 illustrates the principle of DWS. The relative phase φ between the incident wave-fronts of the two interfering beams is averaged over each segment of the quadrant photo diode, and it is compared with the value computed for the other segments, as given by

(1) $$\begin{array}{ccc} {\mathrm{DWS}}_{h} & = & \varphi_{\mathrm{left}}-\varphi_{\mathrm{right}}\\ {\mathrm{DWS}}_{v} & = & \varphi_{\mathrm{top}}-\varphi_{\mathrm{bottom}} \end{array}$$

In this equation, $\varphi_{\mathrm{left}}$ and $\varphi_{\mathrm{right}}$ are averages of the relative phase between the two incident wave-fronts on the photo diode segments A+C and B+D shown in Figure 2. Likewise, $\varphi_{\mathrm{top}}$ and $\varphi_{\mathrm{bottom}}$ correspond to the phase averages on segments A+B and C+D. The indices *h* and *v* indicate the horizontal and the vertical misalignment that are caused by test-mass tip and tilt, respectively. To increase the sensitivity, the variation of the DWS signal must be increased with respect to the test-mass rotation angle θ. This relation is given by

(2) DWS(θ)=mθ.

The sensitivity factor *m*, typically a few 1000 rad/rad, is very roughly given by the ratio of the beam size, ω, over the laser wavelength, $\frac{\omega }{\lambda }$, but it depends in detail on the beam shape and the interferometer geometry. IFOCAD computes it numerically taking these factors into account. The DWS signal is limited to a few hundred microradian before contrast is lost or the sign reverses. To sense a wider rotation range the differential power sensing (DPS) is applied, which provides information about the position of the beam centroid on the photo diode by comparing the average power ${\bar{P}}_{i}$ on the different segments of a quadrant photo diode, as described by

(3) $$\begin{array}{ccc} {\mathrm{DPS}}_{h} & = & \frac{{\bar{P}}_{\mathrm{left}}-{\bar{P}}_{\mathrm{right}}}{{\bar{P}}_{\mathrm{left}}+{\bar{P}}_{\mathrm{right}}}\\ {\mathrm{DPS}}_{v} & = & \frac{{\bar{P}}_{\mathrm{top}}-{\bar{P}}_{\mathrm{bottom}}}{{\bar{P}}_{\mathrm{top}}+{\bar{P}}_{\mathrm{bottom}}}. \end{array}$$

**Figure 3:**
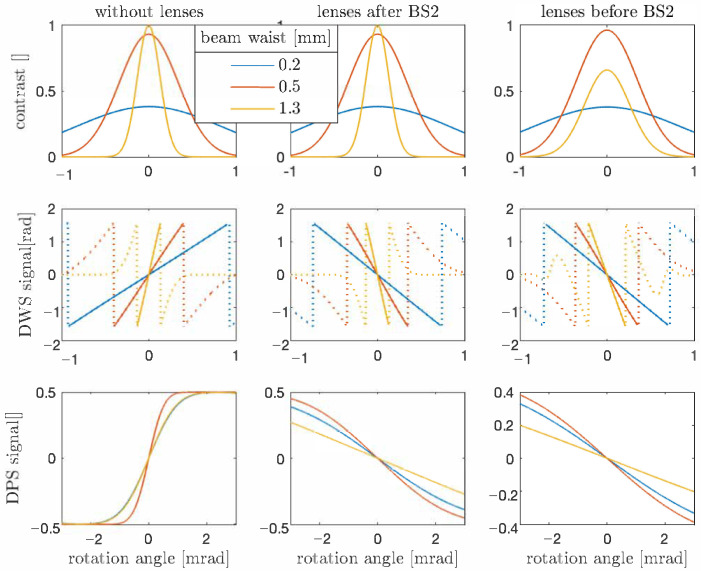
The impact of lenses and also the beam waist size on the interferometric signals. The contrast and the slope of the DWS signal increase by increasing the waist size, whereas the slope of DPS signal depends on the beam spot size on the photo diode. Furthermore, implementing lenses in the setup reduces the beam walk on the photo diode, which results in flatter slope of the DPS signal that allows sensing a wider rotation range. The dotted trajectory in the DWS signal cannot be used for rotation measurement.

The level of the beam walk on the photo diode and consequently, the sensitivity of the DPS signal with respect to the test-mass rotation angle θ is determined by the position and the focal length of the applied lenses. Perfectly aligned beams when the rotating test-mass is imaged on the photo diode center result in a vanishing DPS signal, whereas a long lever arm without any imaging results in a rapid change of the DPS signal with respect to θ, which does not allow for sensing a wide dynamic range. Besides the lens focal length and position, the waist size of the input laser beam, and consequently the spot radius of the incident beam on the photo diodes also influences the interferometric signals. According to the relation $\pi \omega^{2}=\lambda z_{R}$, the Rayleigh range $z_{R}$ depends on the square of the beam waist radius ω, and hence it is shorter for smaller input beams, for which the unequal arm length leads to a stronger loss of contrast. As illustrated in Figure 3, this is the case for the setup without lenses and lenses placed after BS2. Applying lenses before BS2 in the setup, which is not optimized for the input beam waist size, results in a different loss of contrast. The waist position of the input beam in our simulation is kept constant, located at the beam-splitter BS1.

To increase the interferometer performance, the focal length of the lenses $f_{1}$ and $f_{2}$, their position $p_{1}$ and $p_{2}$, and the waist size of the input laser beam ω must be selected properly. In our proposed interferometer setup these parameters are determined by applying an optimizer to the simulated setup, as elaborated below.

The setup is implemented in IFOCAD [14], an in-house C/C++ based simulator for interferometers, which includes an optimization function, called minimizer. This function minimizes a single real function $F(x_{1},\cdots ,x_{i},\cdots ,x_{n})$ of *n* real, continuous parameters $x_{i}$ by varying $x_{i}$ according to nonlinear optimization algorithms.

In our interferometer setup the minimizer was applied such that the following outputs are maximized:

1. The slope of the DWS signal with respect to the rotation angle θ, which is defined by $m=\left[\mathrm{DWS}(\theta )-\mathrm{DWS}(0)\right]/\theta$, for θ→0.
2. The domain of the DPS signal, defined by the interval $\left[\theta_{1},\theta_{2}\right]$, where the slope of the DPS signal with respect to θ is bigger than a given threshold value, that is, 50 $\frac{1}{\mathrm{rad}}$.
3. The domain of the contrast for the DWS signal, defined by the interval $\left[\theta_{3},\theta_{4}\right]$, where the contrast is bigger than a given threshold value, that is, 20%.

These parameters are combined in a single figure of merit function *F* to be minimized as below.

(4) $$F=\left\{\begin{array}{cc} \frac{1}{\left|m\right|}\cdot \frac{1}{|\Delta_{1}|}\cdot \frac{1}{|\Delta_{2}|}, & \mathrm{or}\mathrm{alternatively}:\frac{1}{\left|m\right|}+\frac{1}{|\Delta_{1}|}+\frac{1}{|\Delta_{2}|}\\ \infty & \mathrm{if}\;m,\Delta_{1}\;\mathrm{or}\;\Delta_{2}=0, \end{array}\right.$$

with $\Delta_{1}=\theta_{2}-\theta_{1}$ and $\Delta_{2}=\theta_{4}-\theta_{3}$. In our simulation $F=\frac{1}{\left|m\right|}\cdot \frac{1}{|\Delta_{1}|}\cdot \frac{1}{|\Delta_{2}|}$ provides better results for the setup with lenses before the BS2, whereas $F=\frac{1}{\left|m\right|}+\frac{1}{|\Delta_{1}|}+\frac{1}{|\Delta_{2}|}$ is better for the setup with lenses after the BS2.

As mentioned before, the resulting signals, that is, the contrast, the DWS signal and the DPS signal depend on the lenses’ focal length $f_{1}$, $f_{2}$, their positions $p_{1}$, $p_{2}$ and the input beam waist size ω. Thus, *m*, $\Delta_{1}$ and $\Delta_{2}$ are implemented as functions of $f_{1}$, $f_{2}$, $p_{1}$, $p_{2}$ and ω, and consequently, $F=F(f_{1},f_{2},p_{1},p_{2},\omega )$, which can be processed by the minimizer. For the setup with lenses after BS2 this function is reduced to $F=F(f_{1},p_{1},\omega )$, because the second lens is redundant and if necessary, it can be positioned in a symmetrical way as lens1.

**Figure 4:**
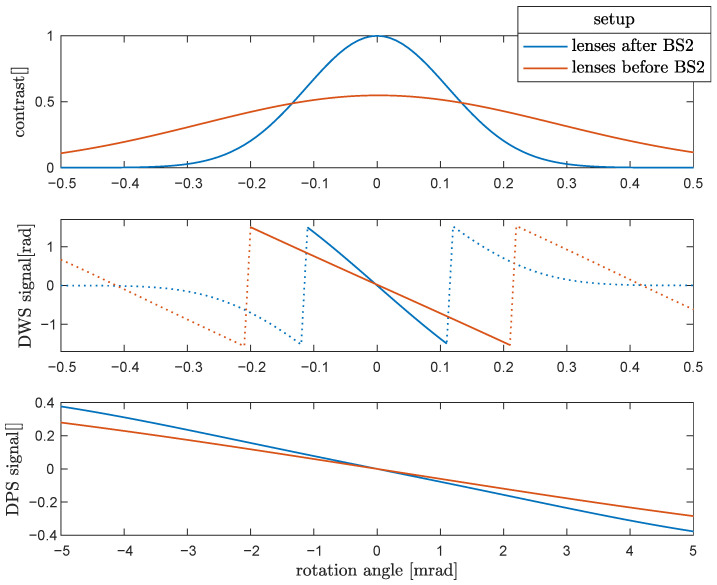
Interferometric signals after optimizing the setup. The setup with lenses after BS2 (blue curves) is preferred because it provides a higher contrast and more sensitive differential wave-front sensing (DWS) and differential power sensing (DPS) signals. The dotted trajectory in the DWS signal cannot be used for rotation measurement.

After calculating the optimized values for $f_{1}$, $f_{2}$, $p_{1}$, $p_{2}$ and ω, one can select and fix focal lengths $f_{1}$ and $f_{2}$ from commercially available lenses close to the optimized values, and also a technically achievable laser beam waist size close to ω. These parameters must be fixed in the interferometer setup, and the minimizer should be applied again to $F=F(p_{1},p_{2})$ to obtain the final position of the lenses. The resulting signals are presented in Figure 4. As shown in the figure, the setup with lenses after BS2 (blue curve) provides a higher contrast and also higher sensitivity of the DWS and the DPS signals. Furthermore, the second lens in this setup is redundant. Therefore, it is the preferred setup.

**Figure 5:**
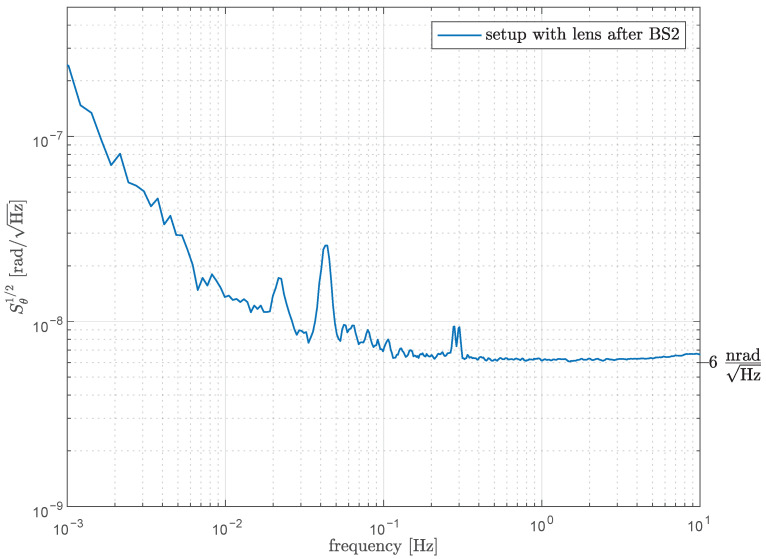
Optimal sensitivity of the test-mass rotation dynamics for the setup with lenses after BS2 calculated by substituting the DWS slope obtained from the simulation with lenses after the beam-splitter in the experiment data reported by Isleif et al [12]. The experiment data was acquired with a very stable quasi-monolithic setup in a vacuum chamber thermalized to a level of 20 μK Hz^−1/2^ at 5 mHz.

The measurement noise around the nominal test-mass position is determined by the DWS signal. From Equation (2) we have

(5) $$S_{\theta }^{1/2}=\frac{1}{m}S_{\mathrm{DWS}}^{1/2},$$

where $S^{1/2}$ are the respective linear spectral densities. Estimation of the potential sensitivity can be derived from a comparable work by Isleif et al [12], which also used DWS measurement together with the deep frequency modulation method in an experimental setting. Although the interferometer conditions are not totally equivalent, for example no lenses are applied in their experiment setup and they use a laser beam with a smaller waist radius (0.5 mm), substituting the DWS slope *m* calculated from our simulation in their experiment data yields a lower limit for a potentially achievable sensitivity. This is 6 nrad Hz^−1/2^ for sub-Hz frequencies and it is plotted in Figure 5 for m=13658.

**Figure 6:**
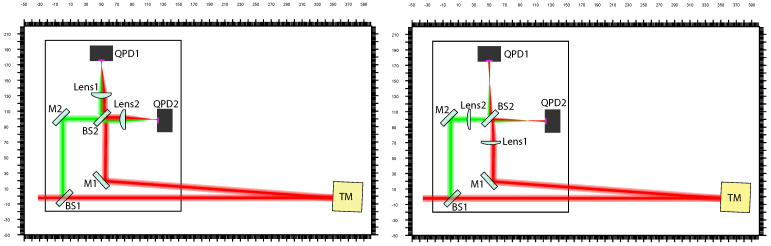
The final interferometer layout after optimization for the setup with lenses after BS2 (**left**) and before BS2 (**right**). The reference beam is highlighted green for sake of comparison. The measurement beam is shown in red and it is illustrated for the case, where the test-mass (TM) is rotated by 5 mrad. The arm length difference between the green and the red path is 571.833 mm. The unit is given in (mm).

In the measurement data by Isleif et al., the interferometer was operating in an optimized environment in a closed vacuum system achieving about $10^{-3}$ mbar and residual temperature variations on the order of 20 μK Hz^−1/2^ above 1 Hz. In addition, it was a quasi-monolithic interferometer with very high stability and intolerance to temperature fluctuations. The interferometer was probably limited by the thermal stability of the fiber collimator, which caused beam jitter at frequencies below 10 mHz. At frequencies above 1 Hz, digitization noise and the noise of the DFM readout algorithm, which was also used, were the limiting noise sources. The interferometer proposed here is very sensitive for an optimal operating point when read out by DWS. However, this deteriorates when the test-mass is tilted and moves the interferometer away from the optimum operating point. The contrast will decrease which will worsen the signal-to-noise ratio. For 0.1 mrad test-mass tilt this is about a factor of two (50% contrast), worsen the sensitivity curve shown in Figure 5 by the same factor to about 12 nrad Hz^−1/2^. For larger test-mass tilts, the interferometer can still be read out by using the DPS signal. The interferometer can brought back to its optimum operating point, for example, by means of a control loop. Predicting the DPS noise from measured amplitude noise spectra of the interferometer is not trivial, since it varies non-linearly with the instantaneous DC power on the photo diode and the voltage at the ADC measurement input. Figure 6 illustrates the final interferometer schemes for both setups. Interferometer details are listed in Table 1, Table 2, Table 3, Table 4 and Table 5.

## 4. Considerations for a Practical Implementation

As the next step we envision an experimental realization for which we will make use of built-in IFOCAD functions that allow the generation of three-dimensional construction details. Based on the geometrical information we will construct a prototype interferometer using low-expansion materials such as Invar and off-the-shelf optical components. In the second step we will advance to template assisted bonding or glueing with UV-curable adhesives. These have proven as suitable techniques in order to reach ultra-low susceptibility to thermal and mechanical disturbances which are a prerequisite for extending high precision sensing into the mHz frequency regime. Accuracies at a level of 25 μm can be obtained by means of template assisted positioning of optical components [17]. This can be improved by the additional use of a coordinate measurement machine, with which the laser beam can be both measured and aligned to the desired direction and position with 10 μ rad angular and 3 μm positional accuracy [18,19]. The use of custom-made, high-quality optical elements will reduce the influence of stray light and unwanted ghost-beams. Furthermore, operating the device in vacuum will reduce the effects of acoustic and temperature noise coupling into the measurements. To further improve the performance at mHz, non-adjustable quasi-monolithic collimators can be used to suppress beam jitter.

To investigate robustness against construction tolerances of the interferometer, all optical components were randomly shifted in the IFOCAD simulation by 25 μm in the two dimensions *x* and *y* of the interferometer plane. Both interferometer layouts show a slight dependency when such a static interferometer misalignment is introduced. The misalignment produces a beam walk on the photo diode, which offsets the DPS and DWS signal. Since we are primarily only interested in relative tilts and not absolute values, this offset is not a major obstacle. Additionally, the offset can be minimized if the photo diode position is readjusted. The parameter that is much more interesting for us, the slope, that is, the sensitivity of DWS and DPS, varies by less than 1%. The analysis of the tolerance to positioning errors of the components, together with the previously obtained accuracies in a comparable experiment, suggests that the realization of the setup is feasible. The required techniques for the implementation are readily available and there exists profound in-house expertise, as demonstrated, for example, in References [20,21].

## 5. Conclusions and Outlook

We proposed a simple Mach-Zehnder type interferometer setup, which is able to sense a wide-range rotational dynamic of a test-mass using DPS and simultaneously, it provides high precision measurement around the test-mass nominal position using the DWS signal. The interferometer has been simulated in IFOCAD and an optimizer algorithm has been applied to find the best focal length and position of the lenses, and also the suitable waist size of the input laser beam. The interferometer can be combined with deep frequency modulation interferometry to simultaneously sense the test-mass translational and rotational dynamics. In a previous DFM experiment, sensitivities at the pm-level have been demonstrated for the translation of the test-mass [12]. We therefore expect to achieve a similar performance with the setup presented here. By combining our findings with the sensing noise measured in the aforementioned DFM experiment, we predict that our setup can provide an angular readout sensitivity of approximately 6 nrad Hz^−1/2^. This is comparable to state-of-the-art devices. The novelty of the scheme presented here is that it simultaneously allows for an extended dynamical range which is typically not the case for other low noise experiments. The simple design and small scale make the interferometer especially suitable for application in torsion pendulum experiments, such as the one at the University of Hannover. The present simulation setup can be easily modified for adoption into other experiments that profit from angular sensing with a large dynamic range and high sensitivity.

## Figures and Tables

**Table 1:** Optical Elements. All units in (mm).

Optical Element	Size	Thickness	Center of the Primary Surface
M1	25.4×25.4	6.35	(52.1, 22.71, 10)
M2	25.4×25.4	6.35	(0, 100, 10)
TM	38×38	38	(350, 0, 10)
BS1	25.4×25.4	6.35	(0, 0, 10)
BS2	25.4×25.4	6.35	(53.29, 99.09, 12.7)

**Table 2:** Input Laser Beam. All units in (mm).

Waist Radius	Wave Length
1.5	1064 × 10^−6^

**Table 3:** Photo diodes. All units in (mm).

Photo Diode	Slit Width	Active Area Radius	Center of the Primary Surface
QPD1	0	3.99	(49.99, 174.77, 10)
QPD2	0	3.99	(122.10, 97.9, 10)

**Table 4:** Setup with lenses after BS2. All units in (mm).

Lens	Focal Length	Thickness	Center of the Primary Surface
lens1	40.1	7.1	(49.9903, 126.65, 10)
lens2	40.1	7.1	(73.9759, 99.17, 10)

**Table 5:** Setup with lenses before BS2. All units in (mm).

Lens	Focal Length	Thickness	Center of the Primary Surface
lens1	75.3	4.4	(52.0941, 67.4, 10)
lens2	75.3	4.4	(21.36, 99.998, 10)

## Data Availability

The data presented in this study can be provided by the corresponding authors upon reasonable request.
